# Persistent “MRI-negative” lupus myelitis-disease presentation, immunological profile and outcome

**DOI:** 10.3389/fneur.2022.968322

**Published:** 2022-10-31

**Authors:** Shambaditya Das, Biman Kanti Ray, Arka Prava Chakraborty, Abhirup Banerjee, Alak Pandit, Gautam Das, Souvik Dubey

**Affiliations:** ^1^Department of Neurology, Institute of Post Graduate Medical Education & Research, Bangur Institute of Neurosciences, Kolkata, India; ^2^Department of General Medicine, Institute of Post Graduate Medical Education & Research, Kolkata, India

**Keywords:** myelitis in lupus, MRI-negative myelitis, MRI-negative lupus myelitis, systemic lupus erythematosus, neuropsychiatric systemic lupus erythematosus, selective tractopathy

## Abstract

**Introduction:**

Myelitis is the least common neuropsychiatric manifestation in systemic lupus erythematosus (SLE). Magnetic resonance imaging (MRI)-negative myelitis is even rarer. Here, we present the largest cohort of MRI-negative lupus myelitis cases to assess their clinical and immunological profiles and outcome.

**Method:**

A single-center, observational study conducted over a period of 5 years (2017–2021) was undertaken to evaluate patients with MRI-negative lupus myelitis for the epidemiological, clinical, immunological, and radiological features at baseline and followed up at monthly intervals for a year, and the outcomes were documented. Among the 22 patients that presented with MRI-negative myelopathy (clinical features suggestive of myelopathy without signal changes on spinal-cord MRI [3Tesla], performed serially at the time of presentation and 7 days, 6 weeks, and 3 months after the onset of symptoms), 8 patients had SLE and were included as the study population.

**Results:**

In 8 of 22 patients presenting with MRI-negative myelopathy, the etiology was SLE. MRI-negative lupus myelitis had a female preponderance (male: female ratio, 1:7). Mean age at onset of myelopathy was 30.0 ± 8.93 years, reaching nadir at 4.9 ± 4.39 weeks (Median, 3.0; range, 1.25–9.75). Clinically, cervical cord involvement was observed in 75% of patients, and 62.5% had selective tract involvement. The mean double stranded deoxyribonucleic acid, C3, and C4 titers at onset of myelopathy were 376.0 ± 342.88 IU/ml (median, 247.0), 46.1 ± 17.98 mg/dL (median, 47.5), and 7.3 ± 3.55 mg/dL (median, 9.0), respectively, with high SLE disease activity index 2,000 score of 20.6 ± 5.9. Anti-ribosomal P protein, anti-Smith antibody, and anti-ribonuclear protein positivity was observed in 87.5, 75, and 75% of the patients, respectively. On follow-up, improvement of myelopathic features with no or minimal deficit was observed in 5 of the 8 patients (62.5%). None of the patients had recurrence or new neurological deficit over 1-year follow-up.

**Conclusion:**

Persistently “MRI-negative” lupus myelitis presents with white matter dysfunction, often with selective tract involvement, in light of high disease activity, which follows a monophasic course with good responsiveness to immunosuppressive therapy. A meticulous clinical evaluation and a low index of suspicion can greatly aid in the diagnosis of this rare clinical condition in lupus.

## Introduction

Systemic lupus erythematosus (SLE) affects multiple neurological systems (1). Neuropsychiatric SLE (NPSLE) encompasses a myriad of symptoms involving the central and/or peripheral nervous system during the disease progression of SLE. In 1999, the American College of Rheumatology (ACR) suggested 19 NPSLE syndromes involving the central or peripheral nervous system. Among these, “myelopathy” is used to specify injury of the spinal cord. It is termed as “myelitis” when spinal cord injury occurs due to inflammatory etiopathogenetic mechanisms (2, 3). It is characterized by neuronal damage resulting in paresis, sensory abnormalities, and autonomic dysfunction (4). Lupus myelitis is the least common presentation of NPSLE. However, its incidence is 1,000 times higher in patients with SLE than in the general population. Thus, this warrants keen attention during the evaluation of patients with SLE. In addition, it remains a serious complication of SLE, often portending a poor prognosis, and is difficult to diagnose and treat (4–6). Diagnostic challenges are compounded when clinically suspected lupus myelitis and the spinal imaging do not correlate (7, 8). The NPSLE case definition of lupus myelopathy does not consider the presence of abnormalities on spinal imaging as a mandatory criterion (3, 8). Magnetic resonance imaging (MRI)-negative lupus myelitis has rarely been reported, and literature pertaining to its clinical presentation, management, and outcome is sparse (1, 7, 8). This study was undertaken to assess the clinical characteristics, biochemical abnormalities, management, and outcome of MRI-negative lupus myelitis in the largest cohort of SLE patients to date (to our best knowledge).

## Materials and methods

**Figure 1:**
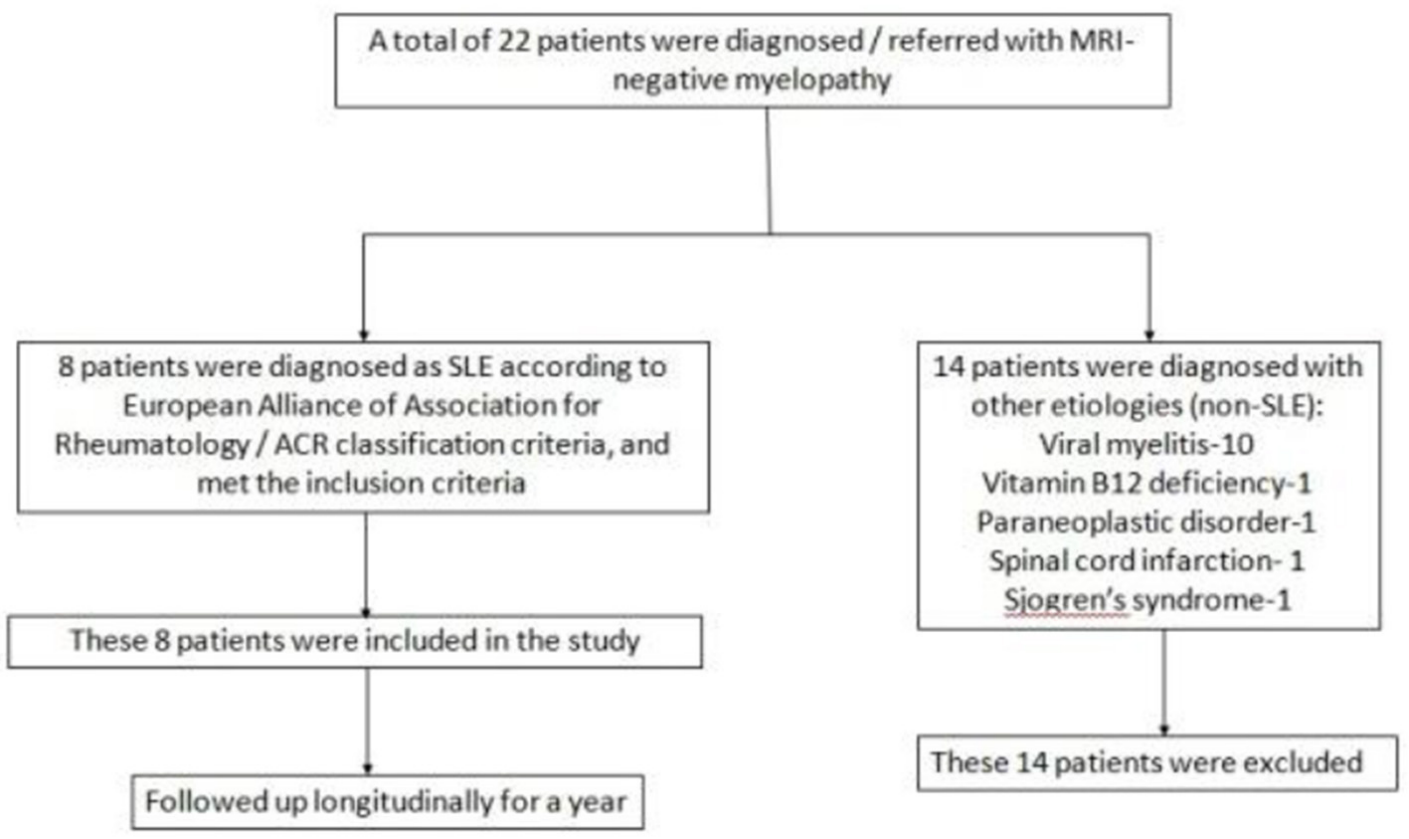
Flowchart showing the study design and enrolment of study population.

In a period of 5 years (2017–2021), 22 patients with MRI-negative myelopathy were either diagnosed or referred to the neuroinflammation clinic of our center (Bangur Institute of Neurosciences, IPGME&R, Kolkata). Among them 8 patients were diagnosed with lupus myelitis. The diagnosis of SLE was confirmed in accordance with the 2019 European Alliance of Associations for Rheumatology /ACR classification criteria. These 8 patients with SLE and MRI-negative myelopathy were included as the study population. A descriptive, observational study with prospective follow-up was conducted to decipher the clinical features, biochemical profile, management, and outcome of MRI-negative lupus myelitis. They were followed-up at monthly intervals with meticulous clinical (symptom analysis and neurological examination) and biochemical assessment for one year. Following a thorough etiological search (Supplementary material S1), out of the rest 14 patients with MRI-negative myelopathy, 10 were diagnosed with viral myelitis. Vitamin B12 deficiency, paraneoplastic disorder, spinal cord infarction, and Sjogren's syndrome were diagnosed in one patient each. Figure 1 shows the study design and enrolment of study population.

Each patient in the study cohort underwent MRI (Siemens 3Tesla MRI machine [Magnetom Verio DOT, 16 channels] using a standard quadrature head coil) imaging of the entire length of the spinal cord (Supplementary material S2) and brain at the time of presentation, followed by repeat spinal cord imaging 7 days later; furthermore, repeat spinal cord imaging was performed at 6 weeks and 3 months following the onset of myelopathic symptoms. An absence of signal change on spinal cord MRI on all four occasions, along with myelopathic evidence defined by the presence of acute/subacute clinical symptoms of motor and/or sensory changes, and/or sphincter dysfunction consistent with spinal cord lesion, corroborated at neurological examination, with exclusion of compressive cord lesion, were considered MRI-negative myelopathy.

They were evaluated under the following major headings: (a) epidemiological- sex, age at diagnosis of SLE, age at onset of myelopathic symptoms, and family history. (b) clinical features- time period between the onset of myelopathic symptoms to nadir, cross-sectional (tracts involved) and longitudinal (spinal cord level) localization, other concomitant central or peripheral nervous system involvement, previous episodes of neurological deficit, evidence of other organ involvement and its temporal relation to myelopathy, and SLE disease activity index-2000 (SLEDAI-2K) score. (c) immunological and radiological features- double stranded DNA (dsDNA) titers (elevated if >100IU/ml), complement levels (decreased if C3 <90 mg/dL, C4 <10 mg/dL); anti-ribonucleoprotein (RNP), anti-ribosomal P protein (Rib-P), anti-Smith antibody (Sm), anti-Sjögren's-syndrome-related antigen A autoantibody (SS-A), anti-Sjögren's-syndrome-related antigen B autoantibody (SS-B) positivity; anti-phospholipid antibodies (lupus anticoagulant, β2-glycoprotein, and anti-cardiolipin) positivity; cerebrospinal fluid (CSF) pleocytosis (cell >5); protein levels (elevated if >45 mg/dL); anti-nuclear antibodies (ANA) positivity; and presence of brain imaging abnormalities. (d) management and outcome immunosuppressive therapy received- functional recovery (medical research council scale for muscle strength grading [MRC]), improvement in objective sensory symptoms, and bladder control (in terms of requirement of urinary catheter), all compared to the neurological status at the time of myelopathic presentation, SLEDAI-2K score at the latest follow-up, and any new-onset neurological deficit or recurrence of neurological symptoms.

The study was performed with the consent of the institutional ethical committee.

### Statistical analysis

Data were summarized using routine descriptive statistics, namely mean and standard deviation for numerical variables that were normally distributed, median and interquartile range for skewed numerical variables, and counts and percentages for categorical variables. Numerical variables were compared between two groups by Student's independent samples *t*-test, if normally distributed, or by Mann-Whitney U test, if otherwise. Fischer's exact test or Pearson's Chi-square test were employed for intergroup comparisons of categorical variables. Analyses were two-tailed and statistical significance level was set at *p* < 0.05 for all comparisons.

## Results

A female predominance was observed (male: female, 1:7) among the eight patients with MRI-negative lupus myelitis. The mean age at diagnosis of SLE and onset of myelopathy were 28.3 ± 8.24 years (median, 26.0; range, 21.75–33.5) and 30.0 ± 8.93 years (median, 28.0; range, 21.25– 37.0), respectively. The mean latency from diagnosis of SLE to onset of myelopathy was 24.5 ± 43.98 months (median, 11.5; range, 3.25– 19.5). The mean time period from onset of myelopathic symptoms to nadir was 4.9 ± 4.39 weeks (median, 3.0; range, 1.25– 9.75). The clinical characteristics suggestive of cervical cord involvement were found to be the most common (75%), followed by dorsal cord involvement, seen in 25% of patients. Selective tract involvement, affecting only the motor and autonomic tracts, was observed in 62.5% of our patients, while the rest had evidence of involvement of all three tracts. Concomitant involvement of other central or peripheral nervous system was observed in 75% of the patients, the most common being polyradiculoneuropathy (37.5%). Myelopathy occurred after other SLE-specific organ involvement in 67.5% of the patients. None of the patients had neurological manifestations prior to the onset of index myelopathic symptoms. Constitutional and mucocutaneous manifestations were seen in all patients (100%); furthermore, there was involvement of musculoskeletal system in 87.5%, hematological and renal involvement in 50% each, and one patient had serosal (pleural) involvement. Among the 4 out of 8 patients with lupus nephritis, two patients denied renal biopsy and the other two had diffuse lupus nephritis (class IV). Those with hematological involvement (4 out of 8), autoimmune hemolytic anemia was seen in one, and leukopenia was found in three and thrombocytopenia was documented in two patients. The mean SLEDAI-2K score at the time of presentation was 20.6 ± 5.9, while it was 0.7 ± 0.95 at the time of most recent follow-up at 1 year. The mean dsDNA, C3, and C4 titers were 376.0 ± 342.88 IU/ml (median, 247.0; range, 177.75–501.5), 46.1 ± 17.98 mg/dL (median, 47.5; range, 22.0– 62.75), and 7.3 ± 3.55 mg/dL (median, 9.0; range, 3.38–10.00), respectively. CSF pleocytosis was seen in 50% patients, ranging from 10–40 cells; increased CSF protein levels were seen in all patients with a mean of 84.9 ± 41.23 mg/dL (median, 65.0; range, 57.25–128.75), and no patient showed CSF ANA positivity. IgG index was raised in 62.5% patients, none had OCB positivity. Among other autoantibodies, Rib-P positivity was observed in 87.5% of patients, and Sm and RNP positivity were observed in 75% of patients. None of the patients demonstrated antiphospholipid antibodies positivity. SS-A positivity was seen in 12.5% patient; while Scl-70, PM-Scl 100, Jo-1, centromere B, nucleosomes, histones, AMA-M2 and SS-B were negative in all patients. Brain imaging abnormalities were detected in 25% of patients. Intravenous pulse methyl prednisolone (IVMP) and cyclophosphamide were instituted in all patients except one, who died due to macrophage activation syndrome (MAS)-related complications prior to completion of IVMP or administration of cyclophosphamide. Two of the eight (25%) patients had an unsatisfactory response to the initial therapy. They were further subjected to rituximab therapy, with one patient receiving plasmapheresis. Among the seven surviving patients, five showed significant improvement with no or minimal neurological deficits. Two patients who received additional rituximab therapy had moderate residual neurological deficits. None of the patients had recurrence or the appearance of new neurological symptoms during the one year follow-up period. The results are summarized in Tables 1, 2.

**Table 1:** Clinical features of our cohort of MRI-negative myelitis in SLE.

**Case**	**Gender**	**Age at diagnosis of SLE(years)**	**Myelopathy**						**Previous episodes of neurological manifestation**	**Other system involved**	**SLEDAI-2K at presentation**
			**Age at onset (years)**	**Time to nadir (weeks)**	**Cross-sectional localization**	**Longitudinal localization**	**Other central or peripheral nervous system involved**	**Temporal relation to other organ involvement**			
1.	Female	21	20	3	Motor, sensory, autonomic	Cervical	Cerebral cortex, Radiculoneuropathy	Preceding	Nil	Constitutional, Mucocutaneous, Musculoskeletal, Hematological	23
2.	Female	26	37	11	Motor, sensory, autonomic	Cervical	Neuropathy	succeeding	Nil	Constitutional, Mucocutaneous, Musculoskeletal, Lupus Nephritis	13
3.	Female	26	27	1	Motor, autonomic	Cervical	Cerebral cortex	succeeding	Nil	Constitutional, Mucocutaneous, Musculoskeletal, Hematological	28
4.	Female	35	37	12	Motor, autonomic	Dorsal	Nil	succeeding	Nil	Constitutional, Mucocutaneous, Musculoskeletal	15
5.	Female	20	20	3	Motor, autonomic	Cervical	Radiculoneuropathy	simultaneous	Nil	Constitutional, Mucocutaneous, Hematological, Lupus Nephritis, Serosal	27
6.	Female	24	25	6	Motor, sensory, autonomic	Dorsal	Neuropathy	succeeding	Nil	Constitutional, Mucocutaneous, Musculoskeletal	15
7.	Male	29	29	2	Motor, autonomic	Cervical	Nil	succeeding	Nil	Constitutional, Mucocutaneous, Musculoskeletal, Hematological, Lupus Nephritis	19
8.	Female	45	45	1	Motor, autonomic	Cervical	Radiculoneuropathy	simultaneous	Nil	Constitutional, Mucocutaneous, Musculoskeletal, Lupus Nephritis	25

**Table 2:** Investigational details, mangement and follow-up of our cohort of MRI-negative myelitis in SLE.

**Case**	**dsDNA titer**	**Complement levels**	**Other antibodies detected positive**	**Anti-phospholipid antibodies**	**CSF Analysis**			**Brain imaging**	**Therapy**	**Functional recovery**	**SLEDAI−2K at last follow-up**	**New-onset neurological defict or recurrence**
					**Pleocytosis**	**Protein**	**ANA**					
1.	Elevated	Decreased	Rib-P	Negative	Present (Mononuclear)	Elevated	Negative	Unidentified bright objects	Steroid, cyclophosphamide, MMF, HCQS	(+)	0	(-)
2.	Elevated	Decreased	RNP, Sm, Rib-P	Negative	Absent	Elevated	Negative	Normal	Steroid, cyclophosphamide, Rituximab, HCQS	(+)	0	(-)
3.	Elevated	Decreased	RNP, Sm, SS-A, Rib-P	Negative	Absent	Elevated	Negative	Normal	Steroid, cyclophosphamide,MMF, HCQS	(+)	1	(-)
4.	Elevated	Decreased	RNP, Sm, Rib-P	Negative	Absent	Elevated	Negative	Normal	Steroid, plasmapheresis, cyclophosphamide, Rituximab, HCQS	(+)	2	(-)
5.	Elevated	Decreased	RNP, Sm, Rib-P	Negative	Present (Mononuclear)	Elevated	Negative	Normal	Steroid, HCQS	-	-	-
6.	Elevated	Decreased	RNP, Sm	Negative	Present (Mononuclear)	Elevated	Negative	Normal	Steroid, cyclophosphamide,MMF, HCQS	(+)	0	(-)
7.	Elevated	Decreased	Rib-P	Negative	Absent	Elevated	Negative	Normal	Steroid, cyclophosphamide,MMF, HCQS	(+)	0	(-)
8.	Elevated	Decreased	RNP, Sm, Rib-P	Negative	Present (Mononuclear)	Elevated	Negative	Unidentified bright objects	Steroid, cyclophosphamide,MMF, HCQS	(+)	2	(-)

ANA, Antinuclear antibody; Anti-dsDNA, Anti-double stranded DNA antibody; HCQS, Hydroxychloroquine; MMF, Mycophenolate Mofetil; RNP, Anti Ribonucleoprotein; Rib-P, Anti-Ribosomal P protein; Sm, Anti Smith antibody; SS-A, Anti–Sjögren's-syndrome-related antigen A autoantibody; SS-B, Anti–Sjögren's-syndrome-related antigen B autoantibody; SLEDAI-2K, Systemic Lupus Erythematosus Disease Activity Index 2000; UBO, Unidentified bright objects.

MRI-negative myelopathy due to etiologies other than lupus had a significantly shorter time to nadir of myelopathic symptoms [6.8 ± 12.74 (median, 1.0; range, 0.5–5.75); p 0.010], lesser concomitant involvement of other central or peripheral nervous system (21.4%; p 0.026) and lesser magnitude of CSF protein elevation [54.6 ± 10.56 mg/dL (median, 54.5; range, 48.75–61.0); p 0.016] as compared to MRI-negative lupus myelitis (Table 3).

**Table 3:** Comparison of baseline clinical and biochemical features between MRI-negative lupus myelitis and MRI-negative myelopathy due to other etiologies.

**Parameters**		**MRI-negative lupus myelitis** **(*n* = 8)**	**MRI-negative myelopathy due to other etiologies (*n* = 14)**	***P*** **-value**
1. Gender (Male: Female)		1:7	8:6	
2. Mean age at onset of myelopathy (years)		30.0 ± 8.93 (median, 28.0; range, 21.25– 37.0)	30.0 ± 12.05 (median, 28.0; range, 19.75– 36.0)	1.000
3. Time to nadir (days)		34.3 ± 32.42 (median, 20.5; range, 8.75– 67.75)	6.8 ± 12.74 (median, 1.0; range, 0.5– 5.75)	0.010
4. Selective tract involvement		62.5%	28.6%	0.187
5. Other central or peripheral nervous system involved		75%	21.4%	0.026
6. Previous episodes of neurological manifestation		Nil	Nil	
7. CSF analysis	(a) Pleocytosis	50%	57.1%	1.000
	(b) Elevated protein	84.9 ± 41.23 mg/dL (median, 65.0; range, 57.25–128.75)	54.6 ± 10.56 mg/dL (median, 54.5; range, 48.75–61.0)	0.016

Illustrative case: A 45-year-old female had complaints of quadriparesis for last 7 days. It started as an acute retention of urine and paraparesis, followed by bilateral upper limbs weakness from the next day, without any sensory and cranial nerve symptoms. She also had history of oral ulcers, malar rash, alopecia, photosensitivity and symmetrical small joint pain and swelling for last 2.5 months along with low grade fever and pedal edema for last 1 month.

Neurological examination revealed diminished muscle power (MRC, Upper limbs: proximally and distally 4-/5; lower limbs: proximally 2/5, distally 3/5), spasticity of all 4 limbs except for hypotonia near both ankle joints, pan-hyper-reflexia except for absent ankle jerk, and bilateral extensor plantar response.

**Figure 2:**
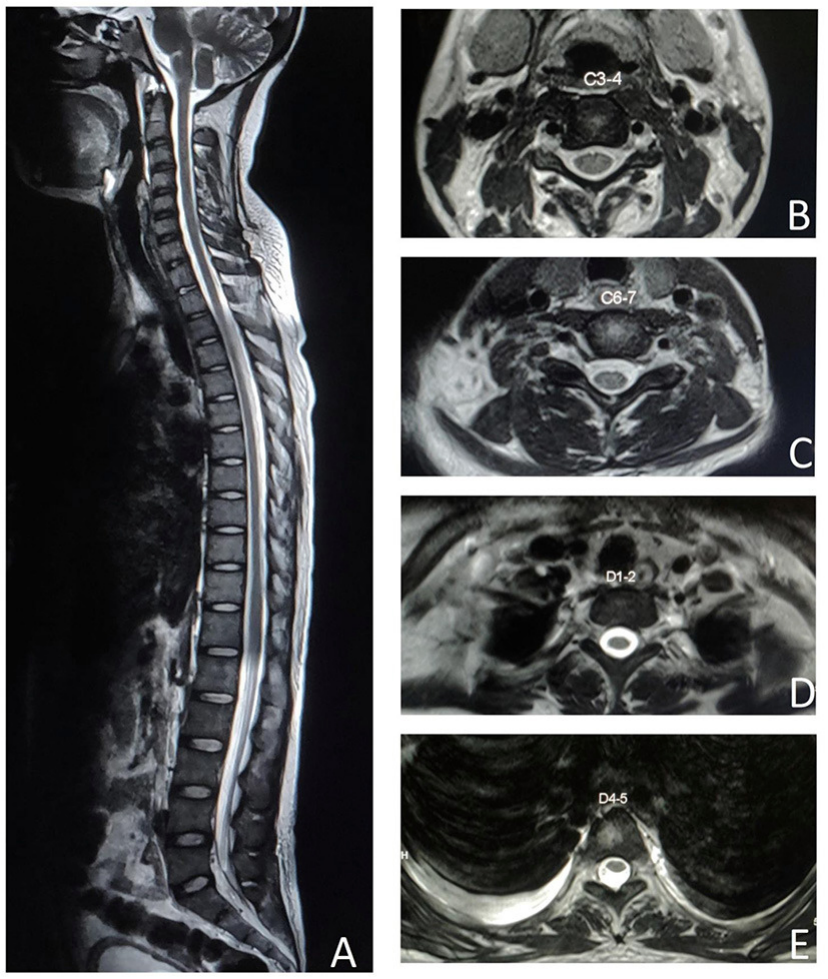
MRI spine T2 weighted image shows no spinal cord signal changes in the sagittal section **(A)**, cervical axial sections at C3–4 **(B)**, C6–7 **(C)**, dorsal axial sections at D1–2 **(D)**, and D4–5 **(E)**.

MRI spine didn't reveal any cord signal change on repeated imaging (Figure 2). Nerve conduction study showed acquired motor axonal polyradiculoneuropathy. CSF analysis had pleocytosis with mildly elevated protein. Biochemical investigations revealed ANA, anti-dsDNA, Rib-P, Sm, and RNP positivity with hypocomplementemia. There was presence of urinary RBC cast with macro-albuminuria on further searching for organ involvement. Patient denied permission for renal biopsy.

She was given 3 days of pulse IVMP therapy (1000 mg/day for 3 days) along with injection Cyclophosphamide (1 gm/month for 6 cycles). Oral Prednisolone was started at 1 mg/kg/day dosing and gradually tapered to 5mg/day by 6 months. Oral Mycophenolate mofetil (2 gm/day) was started following completion of Cyclophosphamide. She had substantial functional recovery in terms motor power (MRC, Upper limbs: proximally and distally 5/5; lower limbs: proximally 4+/5, distally 4/5) and bladder control at the end of 1 year.

## Discussion

Myelitis, which is considered a serious complication of SLE, is one of its least common neuropsychiatric manifestations, occurring in 1–2% of patients with SLE. This may be due to the inherent diagnostic and therapeutic challenges and the increased risk of morbidity and mortality (4, 6). The diagnosis of lupus myelitis is even more obscure in the absence of correlation with imaging (9). Previous observations have suggested that lupus myelitis is the presenting manifestation in nearly half of the patients (6, 10). However, for unknown reasons, our observation suggested that MRI-negative lupus myelitis often occurred after (62.5%) the evidence of other SLE-specific organ involvement. The mean age for lupus myelitis varied from 25–42 years in previous studies (6, 11, 12). A similar predilection toward young adults was also observed in our cohort. Patients with MRI-negative myelitis were predominantly females (87.5%), in line with previous observations and a female predilection for SLE in general (13).

Birnbaum et al. classified lupus myelitis into gray matter and white matter myelitis. Gray matter myelitis is hyperacute and rapidly deteriorates to clinical nadir within 6 hours. It has a severe clinical presentation, with flaccidity and hyporeflexia, and is often monophasic. It more frequently presents with LETM and significant CSF abnormalities. It often occurs in the background of severe systemic inflammation, with a high SLEDAI-2K score, dsDNA titers, and β2-glycoprotein positivity. It is poorly responsive to immunosuppressive therapy and often results in incomplete or poor recovery. White matter myelitis, on the other hand, is characterized by spasticity and hyperreflexia, and the clinical nadir is not reached until 72 hours. It has lower dsDNA positivity. It is more responsive to immunosuppressive therapy and usually has a good prognosis. It is more likely to meet the neuromyelitis optica spectrum disorder criteria and has a higher recurrence rate and lupus anticoagulant positivity (2, 4, 11).

Similar to previous observations, our patients also presented with symptoms related to the involvement of the bilateral motor, sensory, and/or autonomic tracts of variable severity and symmetricity (2, 4). However, it is interesting to note that the majority of our patients (62.5%) had selective tract involvement along the centro-anterior cord, affecting the motor and autonomic tracts, suggesting a predominantly white matter myelitis, according to Birnbaum classification. White matter myelopathy with selective tractopathy has classically been described in few conditions such as multiple sclerosis, paraneoplastic myelopathy, and vitamin B12 deficiency (1, 14). Thus, lupus myelitis, especially in those with lack of correlation with imaging, might be an important consideration in tract-specific white matter myelitis (1).

The proposed pathogenesis for lupus myelitis includes: (i) mechanisms related to anti-phospholipid antibodies, especially β2-glycoprotein, that may lead to thromboembolic effect on microcirculation of spine, or it may interact with certain spinal cord antigens leading to “co-operation between antibodies” and aquaporin-4 (AQP-4) synthesis induction, or may have direct cytotoxic effects. (ii) small vessel vasculitis leading to cord ischemia and necrosis (better explains longitudinal extensive transverse myelitis [LETM] in SLE). (iii) change in blood-brain barrier (BBB) due to complex interplay of overlapping autoantibodies; and (iv) co-clustering of various intertwining pathophysiological mechanisms (cord inflammation, venous hypertension, and cord ischemia) resulting in hemodynamic compromise (1, 2, 4, 6, 10–12, 15).

Lupus myelitis has been seen to develop even during the stages of low disease activity in 1/3rd of the patients (2, 4, 11). However, all patients with MRI-negative lupus myelitis in our cohort showed high disease activity at the onset of myelopathy. CSF analysis for lupus myelitis can vary. It can range from normal (20–33%) to marked pleocytosis, increased protein levels, and hypoglycorrhachia, mimicking bacterial meningitis (2, 4, 11, 12). All our patients had increased CSF protein, and half of them showed pleocytosis (all mononuclear predominant); however, none had more than 50 cells. Rib-P is considered the best biomarker for the diagnosis of NPSLE, and it strongly correlates with NPSLE. Both Rib-P and Sm have been implicated in BBB dysfunction and subsequent aberrant immune downsignaling (16). In our study, we observed high frequencies of antibodies against Rib-P (87.5%), Sm (75%), and RNP (75%). This is a much higher frequency than that of SLE, raising speculations regarding their possible pathogenetic association and causative implications in MRI-negative lupus myelitis. Thus, it may be conjectured that this variant of lupus myelitis is probably an accomplishment of systemic inflammation associated with lupus. The absence of anti-phospholipid antibodies in all patients undermines their role in etiopathogenesis.

Classically, lupus myelitis is acute in onset and progresses to its maximum clinical severity within hours to months (4, 17). Evolution of MRI-negative lupus myelitis in our patients was almost always subacute. The most noteworthy observation was the absence of hyperacute to acute presentation in our cohort, contrary to previous literature, where it has been frequently observed (2, 4, 11, 17). Clinically, the cervical region was the most commonly affected site in our cohort, differing from previous notions of frequent thoracic segment involvement in lupus myelitis. It has been argued that inherent vascular anatomy could be responsible for this thoracic cord predilection (4, 6, 10, 11, 13). The absence of propensity for thoracic cord involvement as well as the absence of hyperacute presentation in our cohort further strengthens our assumption of a lower likelihood of vascular insult-induced myelopathy in this subtype.

Lupus myelitis was commonly associated with concomitant involvement of other neurological systems in our cohort (75%). This value was much higher than that reported in previous studies (13). Associated central and peripheral nervous system manifestations were observed, with the latter being more common. Axonal polyradiculoneuropathy was the most commonly associated condition in our cohort, in line with its previously noted common occurrence in SLE (18).

MRI of the spinal cord with gadolinium contrast administration is considered to be the most sensitive test for the assessment of myelopathy (7). Negative spinal cord imaging is not an unusual phenomenon during the evaluation of clinically suspected acute-to-subacute myelopathy (7–9). As many as 1/5th of patients with myelopathy may not be supported by an obvious lesion on cord imaging (7). This is more commonly seen in idiopathic transverse myelitis (5%), paraneoplastic myelopathy (35%), myelin oligodendrocyte glycoprotein antibody-associated disease (MOGAD), and GFAP-IgG, glycine, and glutamic acid decarboxylase-65 receptor-associated myelopathy. Spinal cord infarction can have an initial negative MRI in 24% of patients, although a hyperacute clinical presentation and absent CSF pleocytosis usually aid in its distinction (7, 9). Sechi et al. postulated that imaging timing (transient lesion being missed on late imaging, or an early imaging failing to detect an evolving lesion) and less sensitivity of MRI (to detect the subtle signal changes related to inflammation of the cord or its surrounding meninges) are the probable reasons for the negative MRI results in MOGAD myelitis (9). An extrapolation to MRI-negative lupus myelopathy may not be far-fetched. However, our cohort underwent repeated imaging with standard sensitivity and negative results. Thus, the absence of imaging abnormalities in MRI-negative lupus myelitis beyond the early stages might suggest a functional disruption in the white matter tracts of the cord without any discernible structural insult. Although two of our patients had evidence of few scattered, tiny white matter changes on brain MRI, primary central nervous system demyelination seemed less likely in light of non-fulfillment of their clinical and biochemical diagnostic criteria (19, 20).

Several novel biomarkers that correlate with neuronal damage have emerged lately. Neurofilament protein levels in blood and CSF have shown promise in assessing the disease onset and progression of nervous system injury, including in Multiple sclerosis (21). Recent evidence has suggested the use of Glial fibrillary acidic protein (GFAP) in detecting subtle injury to CNS (22). The use of these potential biomarkers may contribute to the diagnostic accuracy in MRI-negative lupus myelitis, wherein conventional structural imaging fails to detect the evidence of pathology. Future research in this direction is warranted.

The combination of intravenous glucocorticoids and cyclophosphamide has been the mainstay treatment for lupus myelitis (2, 4, 23). In our cohort, 5 of the 8 patients showed significant improvement with intravenous glucocorticoids and cyclophosphamide therapy. One patient succumbed to MAS in the immediate acute phase just following the initiation of intravenous glucocorticoids. Remaining 2 out of the 8 patients failed to show any significant improvement following initial glucocorticoid and cyclophosphamide administration. Owing to its proposed role in refractory cases (2, 4, 23), plasmapheresis was instituted in one of the two non-responder patients (other patients did not consent to it). Mild improvement in myelopathic features was observed following plasmapheresis. Both patients were further administered rituximab. Clinical improvement was documented in both patients at the subsequent follow-up; although, residual disability persisted. Historically, nearly more than 1/3rd of patients with lupus myelitis have a good prognosis with full recovery or minimal sequelae with appropriate therapy, while about 2/3rd patients suffer from moderate-to-severe disability. LETM has worse prognosis compared to acute transverse myelitis. The previously described poor prognostic factors include clinically severe deficits at onset, need for urinary catheterization, increased number and extension of spinal cord lesions (≥ 4 segments), CSF abnormalities, failure to add cyclophosphamide in a timely manner, and absence of hydroxychloroquine therapy (2, 4, 6, 10, 11, 17). An assessment of the prognostic factors of MRI-negative lupus myelitis in our cohort can be biased due to the small number of patients. However, note must be taken of the fact that both of our patients with residual disabilities on follow-up had a more subacute to chronic evolution of myelopathy in comparison to patients with better functional recovery. The risk of recurrence has been reported to be 18–50%, with at least one episode recurring within a year, despite optimal therapy. None of our patients experienced relapse during the 1-year follow-up period. Positivity to AQP-4 and SS-A/Ro, which are known to increase the risk of recurrence, was absent in our cohort (2, 4, 6, 10).

All our patients with MRI-negative lupus myelitis had a more indolent course, less severe presentation with upper motor neuron (UMN) spasticity and hyperreflexia, milder CSF abnormalities, and relatively good responsiveness to immunosuppressive therapy, which was comparable to the manifestations of white matter myelitis. However, it shared some features with gray matter myelitis. It was monophasic, with a fever prodrome, occurring in light of high disease activity with increased dsDNA titers. Hence, we propose a new subtype of white-matter myelitis in lupus, “MRI-negative myelitis with selective tract involvement,” that occurs in light of high disease activity, often with Rib-P protein positivity, and follows a similar indolent, monophasic course with good responsiveness to immunosuppressive therapy. Although the proposed new phenotype of lupus myelitis shares major similarities with white matter myelitis, the absence of all clinical and biochemical characteristics of gray matter myelitis must not be considered as a rule.

It may be emphasized that often the milder symptoms of myelopathy can be misinterpreted. Mild paresis may be attributed to generalized weakness from the burden of systemic illness. UMN-related bladder symptoms following a cord injury share great similarity with symptoms of urinary tract infection, and sensory symptoms are often vague and non-specific (14, 24, 25). The diagnostic dilemma of myelitis becomes compounded in the absence of correlating MRI findings (8, 26). Thus, diagnosis of MRI-negative lupus myelitis is often difficult, and only meticulous history taking and clinical examination with a low threshold of suspicion can help identify this entity.

Although there is a scope for selection bias due to prior diagnosis of SLE in 5 out of 8 patients in our cohort, the strength of the study lies in the sizeable number of this relatively rare condition of MRI-negative lupus myelitis patients included in the study population.

## Conclusion

MRI-negative lupus myelitis may be an under-reported entity owing to the absence of correlating radiological findings. A high resolution MRI spinal cord imaging with appropriate sequences is essential before its attribution as MRI-negative myelitis. The clinical features of lupus myelitis can mimic other commonly encountered complications of SLE and pose a diagnostic dilemma. High clinical suspicion and meticulous clinical evaluation are mandated for diagnosis. It is mostly associated with high disease activity and a monophasic course. It should be emphasized that timely identification of this complication is of paramount significance, as most cases respond well to appropriately chosen immunosuppressive therapy.

## Data availability statement

The original contributions presented in the study are included in the article/Supplementary material, further inquiries can be directed to the corresponding author.

## Ethics statement

The studies involving human participants were reviewed and approved by Institute of Post Graduate Medical Education & Research, Kolkata, India. The patients/participants provided their written informed consent to participate in this study. Written informed consent was obtained from the individual(s) for the publication of any potentially identifiable images or data included in this article.

## Author contributions

SDa: conceptualization-lead, data curation-lead, formal analysis-equal, investigation-equal, methodology-equal, resources-equal, supervision-equal, visualization-equal, statistical analysis-equal, writing—original draft-lead, and writing—review and editing-lead. BR: conceptualization-equal, formal analysis-equal, investigation-equal, methodology-equal, resources-equal, supervision-equal, visualization-equal, and writing—review and editing-equal. AC and AB: data curation-equal and writing—review and editing-equal. AP: conceptualization-equal, supervision-equal, visualization-equal, and writing—review and editing-equal. GD: supervision-equal, visualization-equal, and writing—review and editing-equal. SDu: conceptualization-equal, formal analysis-equal, investigation-equal, methodology-equal, resources-equal, supervision-lead, visualization-equal, and writing—review and editing-lead. All authors agreed upon the final form of the manuscript before submission.

## Conflict of interest

The authors declare that the research was conducted in the absence of any commercial or financial relationships that could be construed as a potential conflict of interest.

## Publisher's note

All claims expressed in this article are solely those of the authors and do not necessarily represent those of their affiliated organizations, or those of the publisher, the editors and the reviewers. Any product that may be evaluated in this article, or claim that may be made by its manufacturer, is not guaranteed or endorsed by the publisher.
