# Supplementary material for: Persistent “MRI-negative” lupus myelitis-disease presentation, immunological profile and outcome
Source: Front Neurol. 2022 Oct 31;13:968322. doi: 10.3389/fneur.2022.968322 (PMC9659815; doi:10.3389/fneur.2022.968322)
Supplement: Supplementary file 1 [file Data_Sheet_1.docx]

SUPPLEMENT 1
Investigations undertaken for etiological search included:

1. Complete blood count, erythrocyte sedimentation rate, renal and liver function, coagulation profile.
2. Human immunodeficiency virus and Hepatitis-B and C serology
3. Anti-nuclear antibodies (ANA), ANA profile (anti-ribonucleoprotein [RNP], anti-ribosomal P protein [Rib-P], anti-Smith antibody [Sm], Scl-70, PM-Scl 100, Jo-1, centromere B, nucleosomes, histones, anti-mitochondrial M2 antibody [AMA-M2], anti-Sjögren's-syndrome-related antigen A autoantibody [SS-A], anti-Sjögren's-syndrome-related antigen B autoantibody [SS-B]), anti-SS-A and anti-SS-B levels
4. Serum angiotensin converting enzyme
5. Vasculitic profile (perinuclear anti-neutrophil cytoplasmic antibodies [p-ANCA], anti-neutrophil cytoplasmic autoantibody [c-ANCA])
6. Paraneoplastic profile (amphiphysin, CV2, PNMA/Ta, Ri, Yo, Hu)
7. C-reactive protein (CRP)
8. Serum vitamin B12, folic acid, homocysteine, methylmalonic acid, vitamin E, copper levels
9. Aquaporin-4 (AQP4), and myelin oligodendrocyte glycoprotein (MOG) antibodies
10. Cerebrospinal fluid (CSF) analysis to detect the presence of cells, protein and glucose levels, oligoclonal band (OCB), IgG index, and neuroviral panel for viral deoxyribonucleic acid (DNA)-polymerase chain reaction [Zoster, Ebstein–Barr, Herpes simplex, cytomegalovirus, adenovirus, enterovirus, coxsackie B virus, and herpes virus 6]
11. Spinal computed tomography-angiography
12. Patients with suspected SLE were subjected to further testing for other SLE-specific organ involvement.
