# Supplementary material for: Persistent “MRI-negative” lupus myelitis-disease presentation, immunological profile and outcome
Source: Front Neurol. 2022 Oct 31;13:968322. doi: 10.3389/fneur.2022.968322 (PMC9659815; doi:10.3389/fneur.2022.968322)
Supplement: Supplementary file 2 [file Data_Sheet_2.docx]

SUPPLEMENT 2
The sequences of MRI spine undertaken included

1. Pre-contrast T1 (TR- 400, TE- 90): sagittal, transverse
2. T2 (TR- 4000, TE- 90): sagittal, coronal, transverse
3. STIR (TR- 4000, TE- 50, TI- 220): sagittal
4. Post-contrast T1 (Fat suppressed): sagittal, coronal, transverse
